# Salt Stress Causes Peroxisome Proliferation, but Inducing Peroxisome Proliferation Does Not Improve NaCl Tolerance in *Arabidopsis thaliana*

**DOI:** 10.1371/journal.pone.0009408

**Published:** 2010-02-24

**Authors:** Shiro Mitsuya, Mahmoud El-Shami, Imogen A. Sparkes, Wayne L. Charlton, Carine De Marcos Lousa, Barbara Johnson, Alison Baker

**Affiliations:** Centre for Plant Sciences, University of Leeds, Leeds, United Kingdom; Purdue University, United States of America

## Abstract

The PEX11 family of peroxisome membrane proteins have been shown to be involved in regulation of peroxisome size and number in plant, animals, and yeast cells. We and others have previously suggested that peroxisome proliferation as a result of abiotic stress may be important in plant stress responses, and recently it was reported that several rice PEX11 genes were up regulated in response to abiotic stress. We sought to test the hypothesis that promoting peroxisome proliferation in *Arabidopsis thaliana* by over expression of one PEX11 family member, PEX11e, would give increased resistance to salt stress. We could demonstrate up regulation of PEX11e by salt stress and increased peroxisome number by both PEX11e over expression and salt stress, however our experiments failed to find a correlation between PEX11e over expression and increased peroxisome metabolic activity or resistance to salt stress. This suggests that although peroxisome proliferation may be a consequence of salt stress, it does not affect the ability of Arabidopsis plants to tolerate saline conditions.

## Introduction

Peroxisomes are eukaryotic organelles which are highly dynamic and pleiomorphic. They possess a single boundary membrane and lack DNA so their protein complement is acquired by post-translational uptake of cytosolically synthesized proteins which possess a suitable peroxisome targeting signal. A few peroxisomal proteins lack a targeting signal of their own but are imported via association with another protein that does contain a targeting signal, so called ‘piggy-back’ import [1]. In mammalian and yeast cells, peroxisomes have been shown to be capable of arising *de novo* from the endoplasmic reticulum [2], [3], [4], and, although formal proof is lacking, this seems likely to be the case in plant cells as well [5].

Despite the ability to be synthesised *de novo*, peroxisomes possess division machinery [6], which is partially shared with mitochondria [7]. The capacity to divide and be segregated to daughter cells may reflect the requirement for peroxisomes to increase their number and volume rapidly in response to particular developmental and environmental signals. For example peroxisome proliferators such as clofibrate cause peroxisome proliferation in rodents [8]. Yeast peroxisomes are induced to proliferate when the organism is switched to growth on a carbon source that requires peroxisomal metabolism, such as fatty acids [9] or methanol [10]. Plant peroxisomes proliferate during post germinative growth, when peroxisomal β-oxidation plays an essential role in the mobilization of storage lipid [11], and also upon transition of dark grown seedlings into the light [12].

The molecular machinery involved in peroxisome proliferation and division has begun to be characterized (reviewed in [13], [14]). The first protein identified as a regulator of peroxisome size and number was PEX11 from *Saccharomyces cerevisiae*. Disruption of the gene encoding this protein resulted in the formation of a few giant peroxisomes per cell and over expression resulted in many small peroxisomes [15], [16]. Subsequently other genes have been discovered which also act to regulate peroxisome size and abundance; Pex25p, Pex27p [17], [18], and Pex31p and Pex32p [19], [20]. Mammals and plants do not have obvious homologues of these latter genes but have expanded members of the PEX11 family. Phylogenetic analysis has suggested that this expansion has taken place since the divergence of the eukaryotic lineage [21]. There are 3 mammalian isoforms: PEX11α, β, and γ which differ in expression pattern and the phenotypic consequences of gene knockout [22], [23]. The α-isoform stimulates peroxisome proliferation in response to metabolic cues, whereas β appears to play a more constitutive role [24].

In Arabidopsis there are 5 isoforms, designated PEX11a-e [25], which fall into 3 subfamilies on the basis of sequence similarity and show different expression profiles [21]. All 5 isoforms were targeted to peroxisomes and resulted in peroxisome elongation and/or proliferation [21], [25], although some differences were observed between cultured cells [25] and the transgenic plants [21]. Additionally Orth et al were able to show that PEX11e could partially complement the *S. cerevisiae Δpex11* mutant, thereby demonstrating (partial) conservation of function [21].

In mammals PEX11 was shown to interact with DLP1 (dynamin like protein) [26] and more recently a complex containing DLP1, Pex11β and FIS1 was characterized from mammalian cells [27]. Recent studies also point to the involvement of dynamin related proteins playing a role in peroxisome division in plants. The *apm1* mutant of Arabidopsis [28] has a lesion in dynamin-related protein DRP3A which results in elongated peroxisomes and mitochondria, suggestive of a failure to complete division. The *fis1a* and *fis1b* mutants, homologues of mammalian FIS1 which acts to tether DRPs to peroxisomal and mitochondrial membranes, also show a similar cellular phenotype [29], [30]. Arabidopsis PEX11 isoforms d and e were shown to interact with Fis1b using Bimolecular Fluorescence Complementation (BiFC), and to be required for targeting of Fis1b to peroxisomes [31]. Thus PEX11 appears to play a role in recruitment of the molecular machinery for peroxisome division and it is tempting to speculate that the more complex PEX11 families of multicellular organisms reflects an increased requirement to modulate and integrate peroxisomal activities in response to endogenous and environmental stimuli. The demonstration that one specific Arabidopsis PEX11 isoform Pex11b mediates light-induced peroxisome proliferation is consistent with this hypothesis [12].

Peroxisomes compartmentalize many metabolic pathways, but a common function is metabolism of reactive oxygen species. Many peroxisomal activities generate superoxide or hydrogen peroxide [32].The presence of catalase and other anti-oxidative enzymes such as superoxide dismutase, ascorbate peroxidase, dihydro- and monohydro- ascorbate reductase, glutathione peroxidase and glutathione reductase act to scavenge ROS produced within peroxisomes. As well as causing damage to many cellular macromolecules ROS also play a role in a wide range of signal transduction processes that include developmental, hormone and stress related responses. Thus maintaining an appropriate temporal and spatial balance of ROS underpins many plant responses. Whilst the precise contribution of peroxisomal ROS metabolism to stress responses is difficult to quantify, given the presence of multiple isoenzymes in different compartments and separate pools of anti-oxidants such as glutathione, ascorbate and α-tocopherol, a number of studies have provided evidence to link peroxisome proliferation with stress conditions. High light intensity [33] ozone, [34], [35] metal stress [36], [37], salt stress [38], and treatment with the herbicide isoproturon [39] or with the hyoplipidemic drug clofibrate [40] have all been reported to increase peroxisome number or modify activity of peroxisome enzymes involved in ROS metabolism (reviewed in[41]).

Many stresses including salt and drought result in abscisic acid (ABA) accumulation, which results in various protective and adaptive responses such as stomatal closure to limit water loss and changes in transcriptional and post transcriptional gene regulation [42]. Microarray studies in Arabidopsis demonstrated a large overlap between salt, drought and ABA induced genes [43]. ABA signaling operates through a variety of second messengers including phospholipid derived molecules, cyclic ADPribose and hydrogen peroxide [44] and with extensive cross-talk with other phytohormone pathways such as ethylene and jasmonic acid (JA) [45]. Hydrogen peroxide transiently inactivates ABI1 and ABI2, protein phosphatases which act as negative regulators of ABA signaling [44].Among the targets of ABA are many transcription factors such as ABI3 [46] and ABI4 [47], which control specific physiological and developmental responses.

The observation that stress increases peroxisome number and activity begs the question is peroxisome proliferation simply a consequence of stress conditions or does peroxisome proliferation result in an accompanying increase in peroxisomal activities and can this provide a protective effect against oxidative stress? In this study we have investigated whether manipulating peroxisome number via over expression of the PEX11e isoform can improve tolerance of Arabidopsis plants to NaCl.

## Results and Discussion

### PEX11e Is an Integral Peroxisomal Membrane Protein and Forms a High Molecular Weight Complex

**Figure 1:**
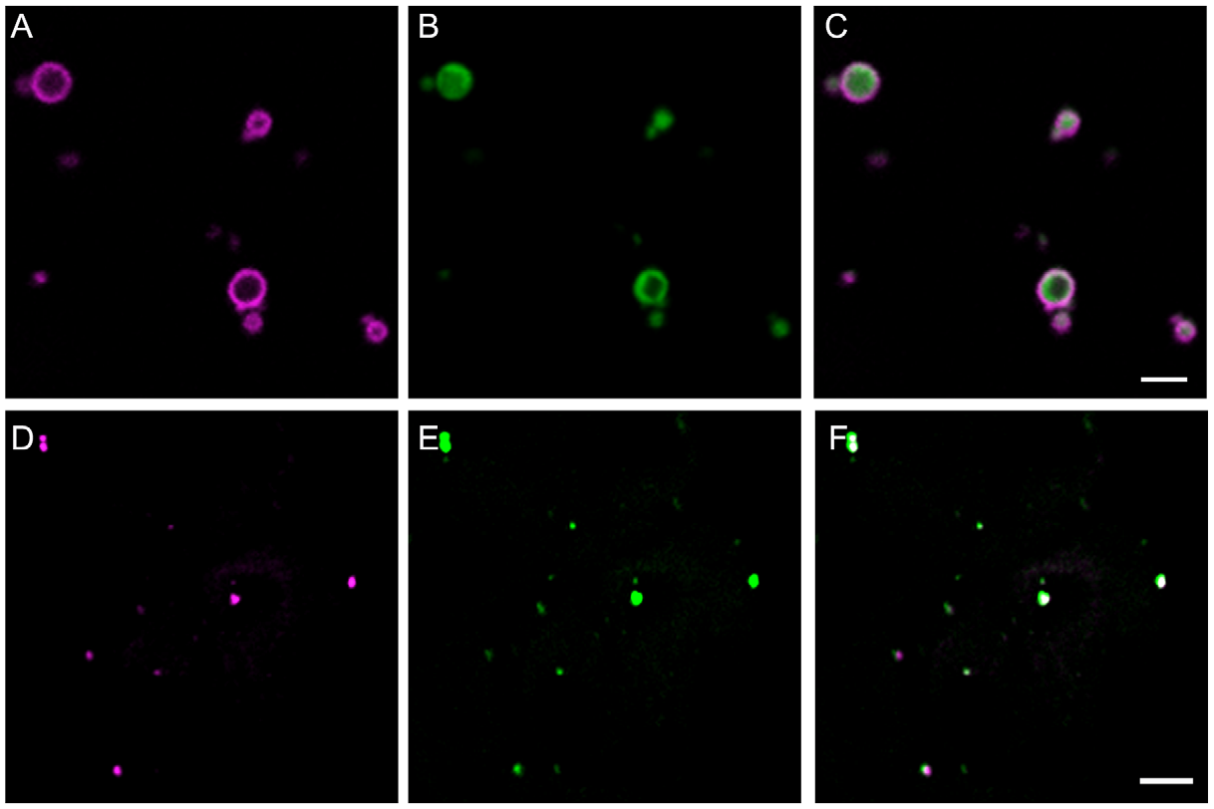
Localisation of PEX11e to peroxisomes. eYFP-PEX11e (panel A, magenta) was transiently co-expressed with the peroxisomal marker CFP-SKL (panel B, green) in tobacco epidermal cells. The two fluorescent markers co-localise in small motile structures 1–2 micrometers in size, typical of peroxisomes (panel C). Arabidopsis suspension cells were immuno-labelled with anti-PEX11 antibody (D, magenta) and anti isocitrate lyase antibody (E, green) a peroxisomal (glyoxysomal) marker protein. The merged figure (F) shows PEX11 in punctate structures containing the glyoxysomal enzyme ICL. Scale bar 2 µm.

Pex11e was selected for this study as it is constitutively expressed at a reasonably high level in most Arabidopsis tissues www.genevestigator.ethz.ch [48]. AtPEX11e was fused at the C terminus of eYFP and expressed by agroinfiltration in the leaf epidermal cells of tobacco plants which stably express the peroxisome targeted reporter CFP-SKL [49]. Figure 1 panel A shows eYFP fluorescence in ring like-structures. Panel B shows the image from the CFP channel which shows that the same structures contain CFP-SKL and the merged image panel C shows that the fluorescence from the eYFP-PEX11 fusion protein surrounds the matrix marker CFP-SKL as would be expected for a peroxisome membrane protein [49]. Occasionally ring type structures can also be seen with matrix markers (an example is visible in panel B.). As peroxisomes sometimes contain matrix inclusions (such as catalase crystals) we hypothesise that such observations represent exclusion of the fluorescent protein from such inclusions. Our results corroborate the findings that N terminally tagged forms of PEX11e target to peroxisomes in transiently transformed BY2 cells [25] and in stably transformed Arabidopsis plants [21] respectively.

Immunofluorescence microscopy was carried out on Arabidopsis suspension culture cells using an antibody raised to a common sequence in PEX11c, d and e. These suspension cultured cells contain very small peroxisomes [49] that contain isocitrate lyase [50]. In Figure 1 panel D punctate structures are detected by the anti-PEX11 antibody that co-localise with the signal obtained with anti-isocitrate lyase (ICL) antibodies (panel E). The merged image panel F shows that these structures are one and the same. As ICL is a peroxisome marker we conclude that native PEX11 is a peroxisomal protein in Arabidopsis as previously reported [21].

**Figure 2:**
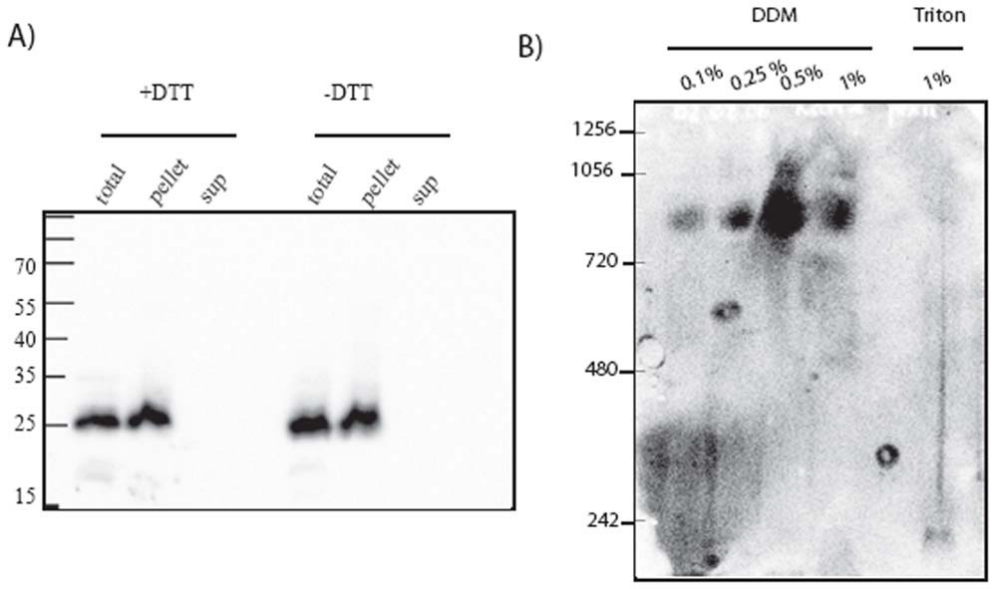
PEX11 is an integral membrane protein that forms a high molecular weight complex. A. Duplicate 100 microgramme mixed light membrane fractions (10,000 g pellet) from Arabidopsis suspension cells was subjected to extraction by 0.1 M sodium carbonate. Soluble and insoluble fractions were separated by SDS-PAGE (with or without DTT) and immunoblotted with PEX11 affinity purified antibody. B. 100 microgrammes of protein from a peroxisome enriched organelle fraction was solubilised in the indicated concentration of detergent and the solubilised proteins were separated on a 3–8% native gel and immnoblotted with anti-PEX11 affinity purified antiserum.

Membranes from Arabidopsis cell culture were treated with alkaline sodium carbonate to remove soluble and peripheral membrane proteins and subjected to SDS PAGE and immunoblotting with anti-PEX11c/d/e antibody. PEX11 was recovered quantitatively in the pellet fraction (Fig. 2A) supporting the notion that it is an integral membrane protein in Arabidopsis [21] [25]. It has been reported that PEX11 in *S. cerevisiae* is peripheral and forms a redox sensitive dimer [51]. We could find no evidence of Arabidopsis PEX11 forming dimers in the absence of DTT (Figure 2A right lanes –DTT).

To explore further whether PEX11 is oligomeric, a peroxisome-enriched organelle fraction from Arabidopsis suspension culture cells was incubated with different concentrations of the detergent dodecyl maltoside, or with 1% Triton X-100 and the soluble fraction (100,000×g supernatant) separated on a native 3–8% polyacrylamide gel. Western blotting of the gel with anti-PEX11c/d/e antibodies revealed that PEX11 forms a complex of approximately 800 kDa in molecular weight in dodecyl maltoside. This complex is unstable in 1% Triton X-100 (Figure 2B). Since the antibodies recognise more than one PEX11 isoform this could be a homo or hetero oligomeric complex as PEX11c, d and e are all expressed in suspension cultured cells [25] (and www.genevestigator.ethz.ch [48]). Even allowing for the presence of detergent molecules, a complex of 800 kDa, must contain multiple copies of PEX11 possibly in complex with other proteins since the predicted monomeric molecular weight of the various PEX11 isoforms is 25.5–25.9 kDa, and the protein(s) run close to their predicted molecular mass on SDS PAGE (Fig. 2A). PEX11e has been shown to homo-dimerise using an in planta split ubiquitin system [52]. A complex containing FIS1, DLP1 and PEX11β could be detected biochemically in mammalian cells [27]. Recently it was shown that the various Arabidopsis PEX11 isoforms could interact with one another and also with FIS1b which acts as a tethering factor for the mitochondrial and peroxisome associated Dynamin related protein DLP1 in mammals [31]. Thus other proteins involved in peroxisome division are also candidate components of the complex observed in Fig. 2B.

### PEX11e Transcripts and Peroxisome Number Are Increased by Salt Stress

**Figure 3:**
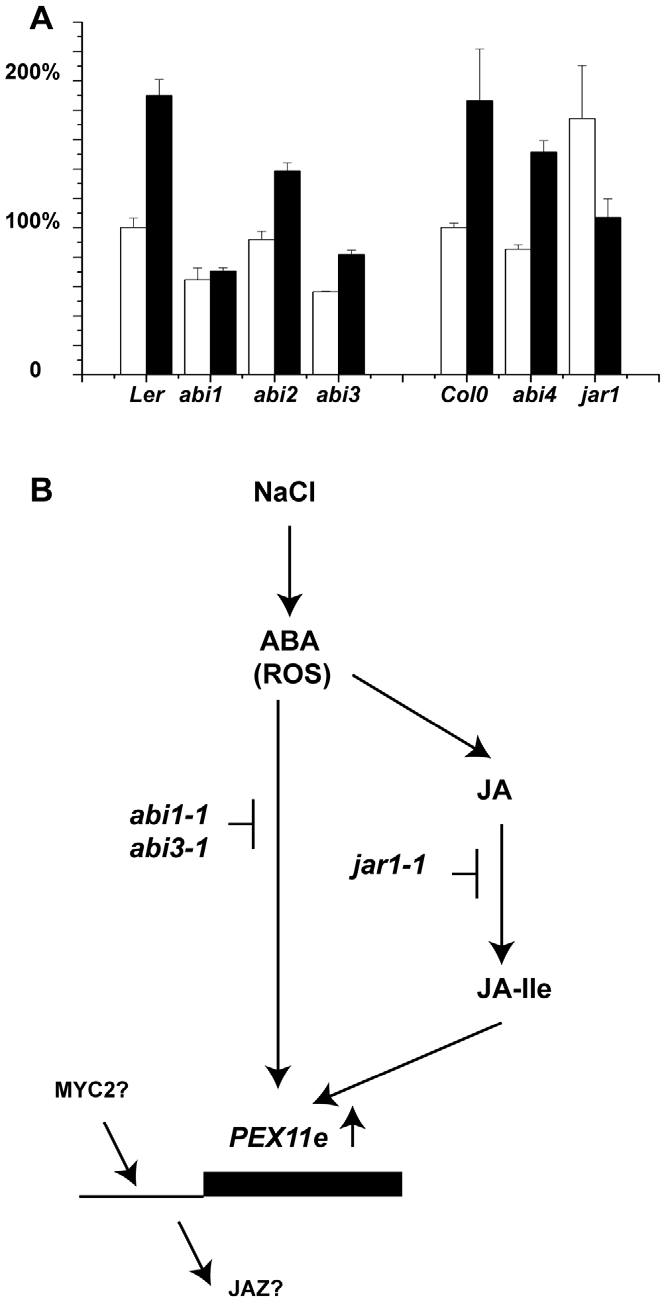
Transciptional response of PEX11e to salt treatment in wild type and signalling mutant backgrounds. A. Pools of 13 day old seedlings of the indicated genotypes were treated with either 400 mM NaCl solution (black bars) or distilled water (white bars) for 4 h. RNA was extracted and the relative levels of expression of *PEX11e* determined by quantitative real time PCR. Untreated control values were set to 1. Data shown are the means of three independent RNA extractions performed on different sets of material grown at the same time with the PCR reactions run in duplicate. B. Model for control of *PEX11e* expression by ABA and JA.

Four members of the rice *PEX11* family have recently been reported to be up regulated by treatment with 200 mM NaCl [53], and we have previously reported the up regulation by salt stress of transcripts for the Arabidopsis peroxisome biogenesis factors *PEX10* and *PEX1* [54] both of which are required for import of peroxisome matrix proteins [55]. Using an identical approach we investigated the changes in steady state levels of *PEX11e* mRNA in response to salt stress in wild type and mutants impaired in abscissic acid and Jasmonate signalling. *PEX11e* transcript was induced two fold after 4 h treatment with 200 mM or 400 mM NaCl (Figure S1). Pools of 13 day old seedlings were treated with 400 mM NaCl or distilled water for 4 h, RNA extracted and transcripts determined by quantitative PCR. All transcripts were normalised relative to Actin2 and the level of expression in the untreated control ecotype set to 100%. In both Landsberg erecta and Columbia ecotypes salt treatment resulted in an approximately 2 fold increase in *PEX11e* transcript (Fig. 3). The increase in expression in response to salt was completely blocked in the *abi1-1* mutant and partially blocked in the *abi3-1*mutant (which is a leaky allele [56]) consistent with a role for abscissic acid in switching on *PEX11e* expression in response to salt. Salt induction still occurred but to a more limited extent in the *abi2-1* and *abi4-103* mutant backgrounds (Fig. 3A). *abi1-1* and *abi2-1* are dominant mutants in type 2C protein phosphatases that are defective in multiple ABA responses [57]. ABI3 and ABI4 are transcription factors that regulate ABA responsive genes. In the *jar1-1* mutant background [58] *PEX11e* is up regulated in the absence of salt and its expression reduced in the presence of salt (Fig. 3), suggesting that salt induction of *PEX11e* may require jasmonate signalling. JAR1 conjugates jasmonic acid to isoleucine to form JA-Ile [59]. JA-Ile promotes association of JAZ transcriptional repressor proteins with SCF^COI1^, the F box subunit of a multi component ubiquitin E3 ligase, leading to their degradation and de-repression of JA-responsive genes [60]. Interestingly, 306 nucleotides 5′ to the ATG of the *PEX11e* gene, in the intron within the 5′ UTR, there is a perfect match to the sequence CGTCA which mediates methyl jasmonate responsiveness of the Barley *LOX1* gene [61] and within the intron (+166) and promoter (+633) is a match to the binding site for MYC2 (ACGTG/CACGT) [62] the key JA transcriptional activator. MYC2 is itself upregulated by both JA and ABA, although ABA induction is independent of ABI1 and most likely occurs by ABA activation of the JA pathway[45]. A possible model for the control of *PEX11e* expression by ABA and JA is presented in Figure 3B. The response of *PEX11e* to salt in the different mutant backgrounds mirrors that of *PEX10* and the β-oxidation enzyme 3-ketoacyl thiolase. [54].

**Figure 4:**
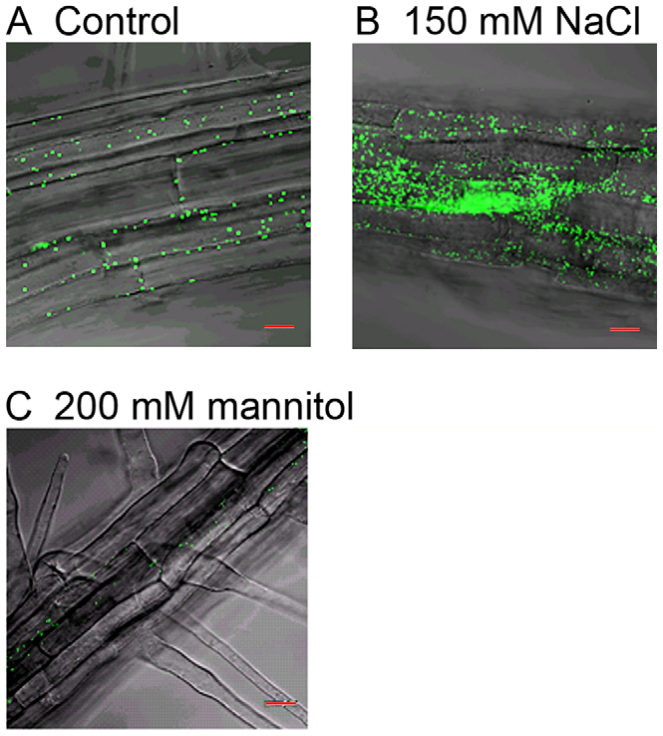
NaCl treatment causes an increase in peroxisome number. Seeds expressing the peroxisome targeted GFP-MFP2 fusion protein (A5 line) were sown on 0.5x MS media supplemented with 1% sucrose and either 150 mM NaCl, 200 mM mannitol or no addition (control). After 5 days the peroxisomes in the roots were imaged by confocal microscopy. Scale bar  = 20 µm.

Since *PEX11e* is up regulated by salt, and up regulation of PEX11e has been reported to induce peroxisome proliferation [21], [31] we tested whether salt stress resulted in peroxisome proliferation. Seedlings of the A5 line which expresses a peroxisomal targeted GFP reporter [63] were grown on 0.5x MS with 1% sucrose supplemented with 150 mM NaCl, or 200 mM mannitol or no supplement for 5 days and GFP in the roots imaged by confocal microscopy. In the control roots peroxisomes were visible (Fig. 4A control). In roots from plants grown on 150 mM NaCl peroxisomes appeared far more abundant (Figure 4B 150 mM NaCl). This effect is due to ionic stress rather than dehydration as peroxisome numbers were not increased in mannitol treated roots (Fig. 4C, 200 mM mannitol), indeed peroxisomes appeared less abundant in mannitol treated roots. Interestingly *PEX1* and *PEX10* transcripts increased in response to salt but not sorbitol treatment [54] suggesting that peroxisomes are responding to the ionic component of salt stress.

### Over Expression of PEX11e Increases Peroxisome Number but Does Not Increase Peroxisome Activity or Tolerance to Salt

**Figure 5:**
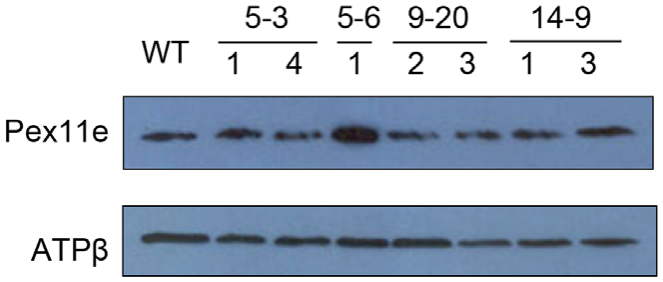
Expression level of PEX11e in transgenic Arabidopsis plants. A crude membrane fraction was prepared from 5 day old seedlings of the lines indicated grown in the dark. Twenty five microgrammes of protein from each line was separated by SDS-PAGE and immunoblotted with anti-PEX11 antibody or anti ATPβ antibody.

Since peroxisome proliferation is induced in salt stress conditions as well as by over expressing PEX11e, we tested the hypothesis that increasing peroxisome number by up regulating PEX11e would provide resistance to salt stress. Columbia ecotype was transformed with a *PEX11e* cDNA construct under the control of the 35S promoter. Three independent transformants (plants 5, 9 and 14) were selected based on high levels of PEX11 expression (Figure S2). T3 (in the case of plant 5 descendents) and T4 (in the case of plant 9 and 14 descendents) were tested for homozygosity (based on 100% kanamycin resistance) and PEX11 expression (based on western blotting). An antibody raised against the β subunit of mitochondrial ATP synthase was used as a loading control. Only one line 5-6-1 consistently showed over expression of PEX11. Five lines were selected for further study, three descended from plant 5 (5-3-1 5-3-4 and 5-6-1) and two each from plants 9 (9-20-2 and 9-20-3)and 14 (14-9-1 and 14-9-3) (Figure 5).

**Figure 6:**
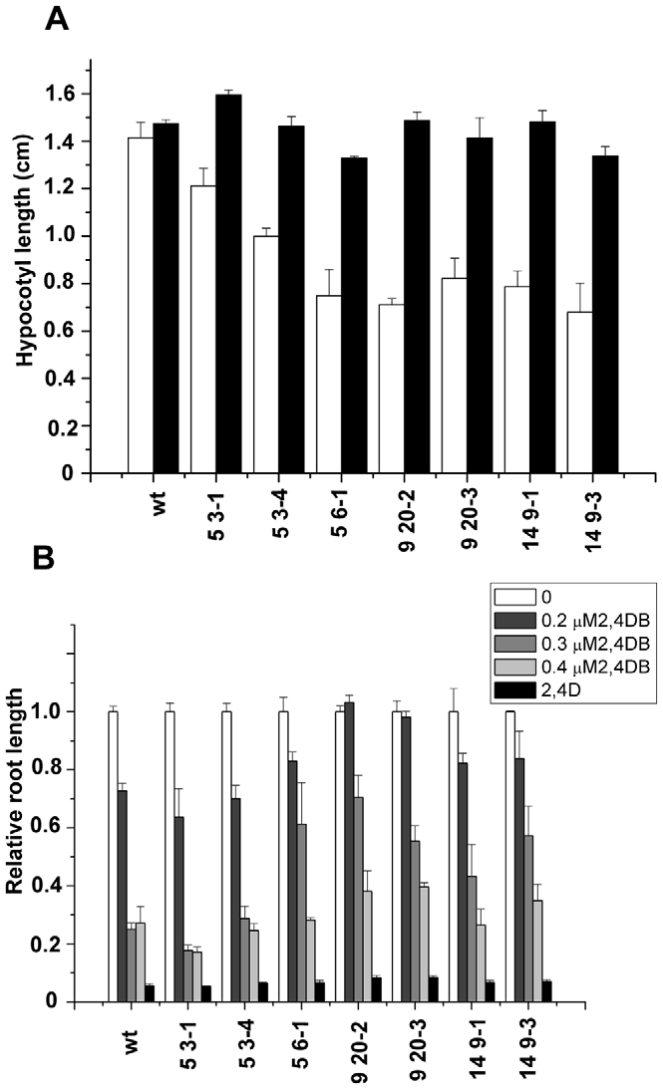
Physiological analysis of PEX11e transgenics. A. Hypocotyl growth in the presence and absence of sucrose. Seedlings were grown for 5 days in the dark on 0.5xMS medium ±1% sucrose and hypocotyl length measured. Results are mean ± SE for 3 replicates of 3–5 seedlings per line. B. Root growth in response to 2,4DB. Seedlings were grown for 5 days in the dark on plates containing 0.5xMS, 0.5% sucrose plus the indicated amount of 2,4DB or 2,4D at 0.05 µg/mL. Results are mean ±SE of 3 replicates of 3–5 seedlings per line normalised to the length of untreated (control) seedlings for each line.

Peroxisome function was tested in the transgenic lines using three different experiments. Firstly the hypocotyl length of 5 day old seedlings grown in the dark in the presence or absence of sucrose was determined. All the lines tested showed a mild reduction in hypocotyl length (50–85%) compared to wild type when grown in the absence of sucrose, which was restored by the inclusion of sucrose in the medium (Fig. 6A). As sucrose dependence can be indicative of impaired β- oxidation, next the growth of the transgenic lines on 2, 4-dichloro-phenoxybutyric acid (2,4-DB) was examined (Fig. 6B). This compound is bioactivated by β-oxidation to the herbicide 2,4D, resulting in inhibition of root growth. All the lines retained sensitivity to 2,4D as expected. Root growth of wild type Columbia was reduced to 20% of the untreated control at 0.3 and 0.4 µM 2,4 DB comparable with previously published results [64]. Lines 5-3-1 and 5-3-4 showed no resistance to 2,4DB whereas lines 5-6-1 9-20-2, 9-20-3, 14-9-1 and 14-9-3 showed increased resistance to 0.3 µM 2,4DB indicative of a partial block in conversion of this compound to 2,4D. These lines also had the shortest hypocotyls when grown in the absence of sucrose. For comparison a strong β-oxidation mutant such as *ped3-4* is completely resistant to 0.8 µM 2,4-DB and, when grown in the absence of sucrose, produces very short hypocotyls that are ca 20% of the length of hypocotyls of plants grown on sucrose [64]. Thus the PEX11e transgenics appear to have a mild β-oxidation deficiency phenotype. This is in contrast to results obtained by Orth et al [21] who saw no sucrose dependence of PEX11 e over expressing lines, although these authors did not report on 2,4DB sensitivity.

Finally, to investigate a β-oxidation independent function, catalase activity was measured in homogenates from leaf material of the transgenic lines but no striking differences were observed between the lines (data not shown). Western blotting was carried out with anti-thiolase antibodies (on dark grown seedlings) and anti-glycolate oxidase antibodies (on green leaf tissue). While lines 5-3-1 and 5-3-4 showed slightly reduced levels of thiolase protein, there were no changes of level of protein correlated with increased expression of PEX11e. Likewise there were no striking alterations of protein level of glycolate oxidase in the transgenic lines.(Figure S3).

**Figure 7:**
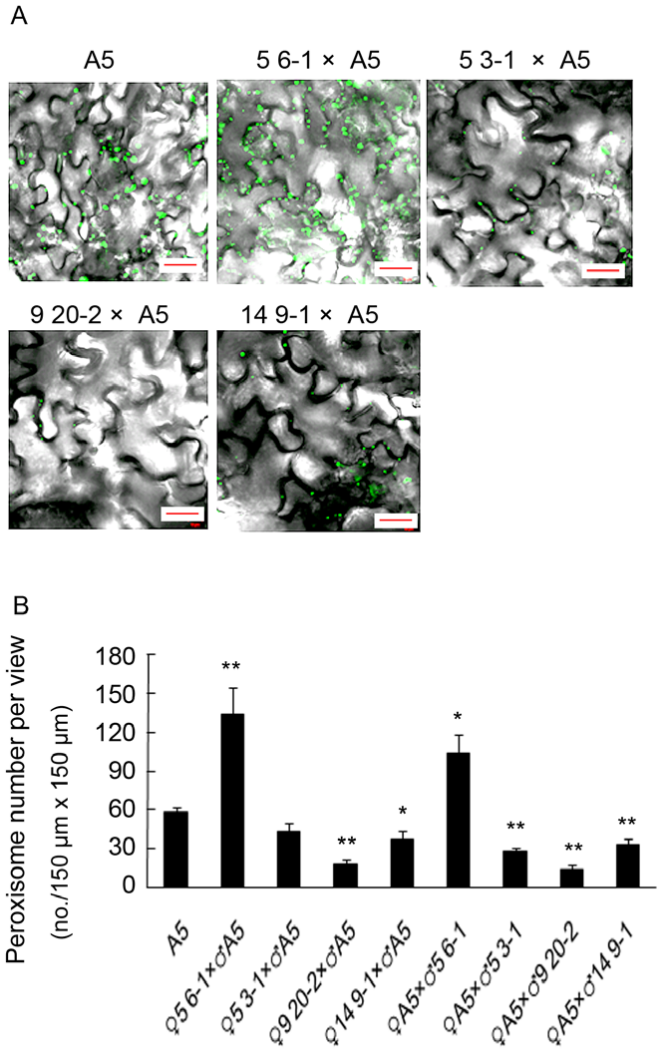
Over-expression of PEX11e results in increased peroxisome number. Reciprocal crosses were made between the PEX11e transgenic lines shown in figure 5 and the A5 line. F1 seed were plated on 0.5x MS supplemented with 1% sucrose, basta and hygromycin to select for both transgenes. Control (homozygous A5 plants) were grown without selection. A. After 2 weeks peroxisomes were observed in the leaves by confocal microscopy. B. Peroxisomes were counted in 10 fields (146.57×146.57 µm^2^) per cross. * and ** represent significant differences from A5 at t<0.01 and t<0.05, respectively (Student's t-test).

Plants of lines 5-3-1, 5-6-1, 9-20-2 and 14-9-1 were crossed to the A5 line as male or female parent and F1 selected on kanamycin plus basta plates to select for both transgenes. Leaves were analysed by confocal microscopy after 14 days and the number of peroxisomes quantified (Fig. 7). Crosses with the 5-6-1 line as male or female parent resulted in an increase in peroxisome number consistent with previous reports for over expression of PEX11e [21], [25]. The other lines when crossed to the A5 line did not result in an increase in peroxisome number compared to the A5 control. Interestingly, the peroxisome number was always higher when the PEX11e transgenic was used as the female parent, compared to the reciprocal cross. A reduction in peroxisome number compared to the A5 line was seen in both reciprocal crosses with the 9-20-2 and 14-9-1 lines. This reduction in peroxisome number along with the loss of PEX11e over expression from descendants of plants selected for high expression may indicate that high levels of PEX11e expression are not well tolerated by Arabidopsis.

**Figure 8:**
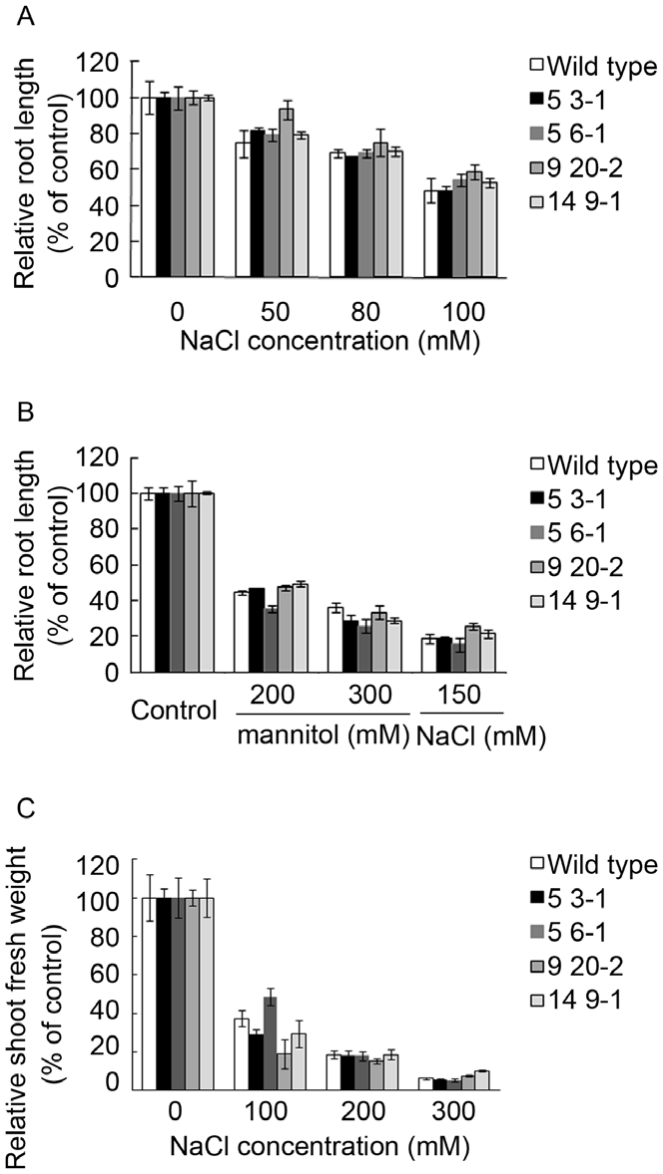
Seedlings with increased level of PEX11e do not show increased resistance to NaCl stress. Seeds from the lines shown in Figure 5 were grown on 0.5x MS medium plus the indicated amount of NaCl or Mannitol. Root length was measured after 5 days and plotted as a % of the unsupplemented (control) value for each line (A and B). After 24 days the fresh weight of the shoots was determined and expressed as a % of the unsupplemented (control) (C). Graphs show the mean and standard error of 3–5 seedlings from each of 3 independent experiments.

To test if any of the PEX11e lines had greater resistance to salt stress seeds were grown on medium supplemented with the indicated amounts of NaCl or mannitol for 5 days, the roots were measured and growth was expressed relative to that of the control. None of the lines tested showed any increased resistance to NaCl (Fig. 8A and B). To test the effect of long term salt stress plants were grown in soil and watered with NaCl supplemented water at the indicated concentrations. After 24 days fresh weight was determined (fig. 8). Again there was no effect on fresh weight.

### Conclusions

Arabidopsis PEX11e is targeted to peroxisomes in tobacco epidermal cells when expressed as a fusion protein with eYFP, and native PEX11c,d,e are peroxisomal in Arabidopsis suspension cells. Pex11c,d,e behave as integral membrane proteins and no evidence could be found for a redox sensitive dimer as has been reported for *S. cerevisae* PEX11[51]. On native gels PEX11 migrates as a large complex. *PEX11e* is up regulated by NaCl stress in an ABI 3- ABI1- and JAR1-dependent manner suggesting ABA and JA signalling act downstream from salt stress to regulate *PEX11e* expression. Ionic but not dehydration stress resulted in an increase in peroxisome number, but increasing peroxisome number by up regulation of PEX11e did not provide increased resistance to salt stress in seedlings or older plants. This is in contrast to studies which have shown that over expression of specific peroxisomal enzymes ascorbate peroxidase [65] and betaine aldehyde dehydrogenase [66] gave enhanced stress tolerance. However the studies reported here did not find any evidence that increased PEX11e abundance actually increased peroxisome activity. The transgenic lines rather showed a mild β-oxidation phenotype in seedlings and no indication of increased catalase activity in leaves. Although increased *PEX11e* expression by salt could provide a mechanism for peroxisome proliferation under salt stress conditions, the hypothesis that the quantity of peroxisomes limits plant stress tolerance proved incorrect and increasing peroxisome number by manipulating peroxisome biogenesis related genes is unlikely to be a useful strategy for increasing salt tolerance.

## Materials and Methods

### Construction and Characterisation of Transgenic Plants

The PEX11e gene was amplified from an Arabidopsis 2 day seedling cDNA library (a gift from Prof Ian Graham, University of York) with the primers (5′ ATGACTACACTAGATTTGACC 3′), and antisense (5′TCTTCAACTTGGGGCGCGATG 3′) and the product cloned into pGEM-T easy and fully sequenced. Overexpression constructions were carried out using the GATEWAY system (Invitrogen). The construct was generated by PCR with two primers: attB1-*At*Pex11-2 (5′-GGGGACAAGTTTGTACAAAAAAGCAGGCTTCACCATGACTACACTAGATTTGACC-3′) and attB2-*At*Pex11-2 (5′-GGGGACCACTTTGTACAAGAAAGCTGGGTCTCATGATTTCTTCAACTTGG-3′) The PCR products flanked by *attB1* and *attB2* (underlined) were recombined into pDONR-207 (Invitrogen).

The intermediate, *At*Pex11e *attL1 –attL2*-containing pDONR-207 vector was recombined with the Gateway enabled destination vector pGD625 [67] containing the 35S promoter and nos terminator which we used for overexpression. Inserts containing clones were verified by restriction enzyme digestion and by sequencing. Arabidopsis plants ecotype Columbia were transformed by the floral dip method [68], selected on kanamycin and checked for overexpression of PEX11e by western blotting. Three T1 plants (lines 5, 9 and 14) were selected for further analysis (Figure S1). Experiments described in this paper were performed on homozygous T4 (lines beginning 5) or T5 (lines beginning 9 or 14) plants which were selected from parents showing high level of expression of PEX11e by western in the previous generation. For confocal analysis reciprocal crosses were made to plants of the A5 line [63] which expresses a peroxisomal targeted GFP-MFP2 fusion protein.

### Antibodies, Polyacrylamide Gel Electrophoresis and Western Blotting

A polyclonal antiserum was raised in rabbit to the peptide VLYLNKAEARDKICRAIQYGSKFLSC corresponding to amino acids 15 to 40 of Arabidopsis PEX11e (Genosphere technologies). This region is highly conserved in PEX11c, d and e, therefore the antibody would be expected to cross react with these isoforms as well. The carboxy-terminal C was added to facilitate affinity purification of antibodies by immobilising the peptide antigen to SulfoLink Coupling Gel (Pierce) according to the manufacturer's standard protocol, yielding affinity purified antibodies with protein concentrations of 0.7 and 0.9 mg ml^−1^. Proteins were separated by standard SDS-PAGE and transferred to nitrocellulose membranes (0.45 µm, Micron Separations, Schleicher&Schuell). Except where indicated, sample volumes loaded on gels were equivalent. Membranes were blocked overnight at 4°C in TBST (50 mM Tris-HCl, pH 7.4, 200 mM NaCl, and 0.1% v/v Tween 20) containing 5% (w/v) non-fat dry milk, then incubated in TBST plus 5% (w/v) non-fat dry milk (TBST-milk) containing affinity-purified PEX11e antibodies at dilutions of 1∶2,000. Incubations with primary antibody were carried out 2 h at temperature room. After four 10-min washes in TBST, membranes were incubated with horseradish peroxidase-conjugated goat-anti-rabbit secondary antibodies (Sigma) for 2 h at 1∶3,000 dilution in TBST-milk. After four10-min washes in TBST, membranes were developed by enhanced chemi-luminescence and the signal was recorded on X-Ray film (Fuji Medical).

### Preparation of Membrane Fractions from Seedlings

Seedlings were ground to a fine powder in liquid N_2_. Homogenisation buffer (50 mM Tris HCl pH 8.2, 2 mM EDTA, 20% v/v glycerol, 1 mM PMSF, 2% protease inhibitor cocktail, 0.5 mM DTT was added at a ratio of 0.1 mL per 0.1 g fresh weight and ground again to produce a slurry. The homogenate was centrifuged 10 min 800 g 4°C, the supernatant carefully removed and centrifuged for 30 minutes at 4°C 100,000 g. the supernatant was removed and the pellet resuspended in 30 microlitre homogenization buffer plus 5 microlitres 10% SDS.

### Preparation of Membrane Fractions from Cell Culture

Cell culture was pelleted and washed twice in PBS and once in homogenisation buffer 50 mM Tris-HCl pH 8.2, 2 mM EDTA, 20% Glycerol, and protease inhibitor tablets (Sigma). The cell pellet was then ground in liquid nitrogen for 10 min, and resuspended in homogenisation buffer at 1 ml/g ground material. This was finally filtered twice through miracloth (Millipore) and spun for 10 min at 3000 g at 4°C. The supernatant was submitted to a second centrifugation at 10000 g for 30 min at 4°C. The pellet was resuspended in homogenisation buffer and the protein concentration was measured with BCA reagent (Pierce) with bovine serum albumen as standard.

### Carbonate Extraction

100 microgrammes of proteins were aliquoted and either left untreated (T) or extracted by 0.1 M Na_2_CO_3_ for 30 min on ice. Aliquots were then spun for 20 min at 4°C. The supernatant was precipitated with 20% TCA for 20 min and pelleted at 14000 g for 20 min. Pellets were resuspended in sample buffer with or without DTT as indicated and loaded on a denaturing gel. As a control thiolase (peroxisome matrix protein) signal was found in the supernatant (not shown) confirming the success of carbonate extraction.

### Native Gel

100 microgrammes of protein from a peroxisome enriched organelle fraction was aliquotted and solubilised in the indicated concentration of detergent (dodecyl maltoside; DDM, or Triton X-100; Triton) for 30 minutes on ice. After a clearing spin of 100,000 *g* for 20 minutes at 4°C, the samples were separated on a NuPAGE 3–8% native gel (Invitrogen) according to the manufacturer's instructions. After transfer to PVDF the membrane was incubated with anti-PEX11 affinity purified antiserum.

### Salt Stress Experiments

Treatment of seedlings, quantitative real time PCR experiments and analysis of data were carried out exactly as described in Charlton et al., 2005. Primers 11-2F

5′-TGA ATT GCT TGG ACG TAT ATC ACT TT -3′and 11-2R 5′-CAC CAA TCT CGA CTG CAC TTG T-3′ were used.

For seedling experiments seeds were grown on 0.5x MS media, supplemented as described in the figure legends. Plates were maintained in a growth room (16 h light 20 °C) for the required number of days before being scanned and root length quantified using the programme Image J. For longer term effects of salt stress seeds were germinated on 0.5x MS agar plates. After 1 week they were transplanted to soil and watered with tap water for a further week. Plants were then watered with tap water supplemented with 100, 200 or 300 mM NaCl for a further 24 days before measuring the fresh weight.

### 2,4DB Resistance and Sucrose Dependence

Experiments were carried out as described [64].

### Confocal Microscopy and Immunofluorescence

AtPEX11e was amplified using Pfx from the AtPEX11 overexpression vector (see previously) using primers AtPEX11-2A (5′ GGGGACAAGTTTGTACAAAAAAGCA GGCTTCCCGCCAATGACTACACTAGATTTGACCAGAG 3′) and AtPEX11-2B (5′ GGGGACCACTTTGTACAAGAAAGCTGGGTCTCAAGGTGTCTTCAACTTGG 3′), cloned into pDONOR207 and subsequently recombined into the plant binary destination vector 35S-eYFP-cassette-Nos::pCAMBIA 1300 [49] resulting in eYFP-AtPEX11e. All vectors were sequenced and verified. *Agrobacterium tumefaciens* was transformed according to the freeze thaw procedure [69]. Transient expression in tobacco leaf epidermal cells was carried out according to Sparkes *et al* [70] where eYFP-AtPEX11e and CFP-SKL were infiltrated at an optical density of 0.1 and 0.04 respectively. Dual expression was imaged using a Zeiss inverted LSM510 using the settings stated in Sparkes et al. [49]. Arabidopsis cell culture was harvested 4 days after subculture, immunofluorescence and subsequent imaging were carried out according to Sparkes et al. [49], where anti-PEX11 antibody (1∶40) and anti-ICL (1∶1000) with Texas red-conjugated goat anti-rabbit IgG (H+L; Molecular Probes; 1∶100) and FITC-conjugated goat anti-rabbit IgG (Sigma Aldrich, 1∶40) respective secondary antibodies were used.

To determine the effect of NaCl on the morphology of peroxisomes, A5 seedlings were grown on 0.5x MS with 1% (w/v) sucrose supplemented with 150 mM NaCl, 200 mM mannitol or no supplement for 5 days and the middle part of the roots was used for confocal microscopy. For numerical analysis of peroxisomes in Pex11-e overexpressing plants, the seedlings of reciprocal crosses between Pex11-e introduced plants and A5 line were grown on 0.5x MS with 1% (w/v) sucrose plates containing 50 µg/ml kanamycin and 10 µg/ml glufosinate ammonium (BASTA) for 2 weeks. Then the abaxial epidermis of leaves was used for confocal microscopy and the number of peroxisomes was obtained from confocal microscope images captured from 150 µm×150 µm.

## Supporting Information

Figure S1 PEX11e is upregulated by salt stress. Seedlings were treated with 200 mM or 400 mM NaCl for 4.5 h then the steady state levels of PEX11e transcripts determined by QPCR.
(1.76 MB TIF)

Figure S2 Primary transformants with increased level of expression of PEX11e. Forty microgrammes of membrane protein from 35 day old primary transformant plants number 5, 9 and 14 were separated by SDS PAGE. Panel A immunoblot with anti-PEX11c/d/e antiserum. Panel B Coomassie protein stain of the same membrane protein fractions.
(0.45 MB TIF)

Figure S3 PEX11e transgenics do not have altered levels of glycolate oxidase or 3-ketoacyl thiolase protein. Western blot analysis of thiolase and glycolate oxidase in total protein extracts (20 µg per lane) from dark grown seedlings (thiolase) and green leaves of 4 week old plants (glycolate oxidase) of the indicated lines. The Ponceau S strained membrane is shown in each case to verify equal loading of the lanes.
(6.98 MB TIF)
