# Supplementary figures and images for: Salt Stress Causes Peroxisome Proliferation, but Inducing Peroxisome Proliferation Does Not Improve NaCl Tolerance in Arabidopsis thaliana
Source: PLoS One. 2010 Feb 24;5(2):e9408. doi: 10.1371/journal.pone.0009408 (PMC2827565; doi:10.1371/journal.pone.0009408)

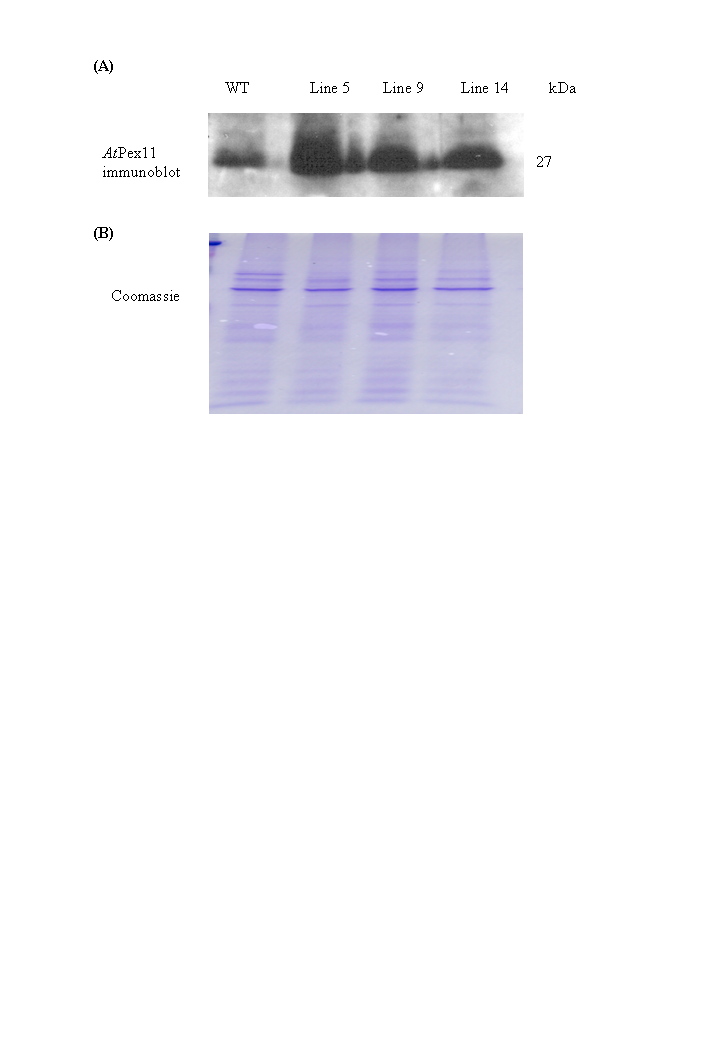

Supplement: Figure S2 — Primary transformants with increased level of expression of PEX11e. Forty microgrammes of membrane protein from 35 day old primary transformant plants number 5, 9 and 14 were separated by SDS PAGE. Panel A immunoblot with anti-PEX11c/d/e antiserum. Panel B Coomassie protein stain of the same membrane protein fractions. (0.45 MB TIF) [file pone.0009408.s002.tif]

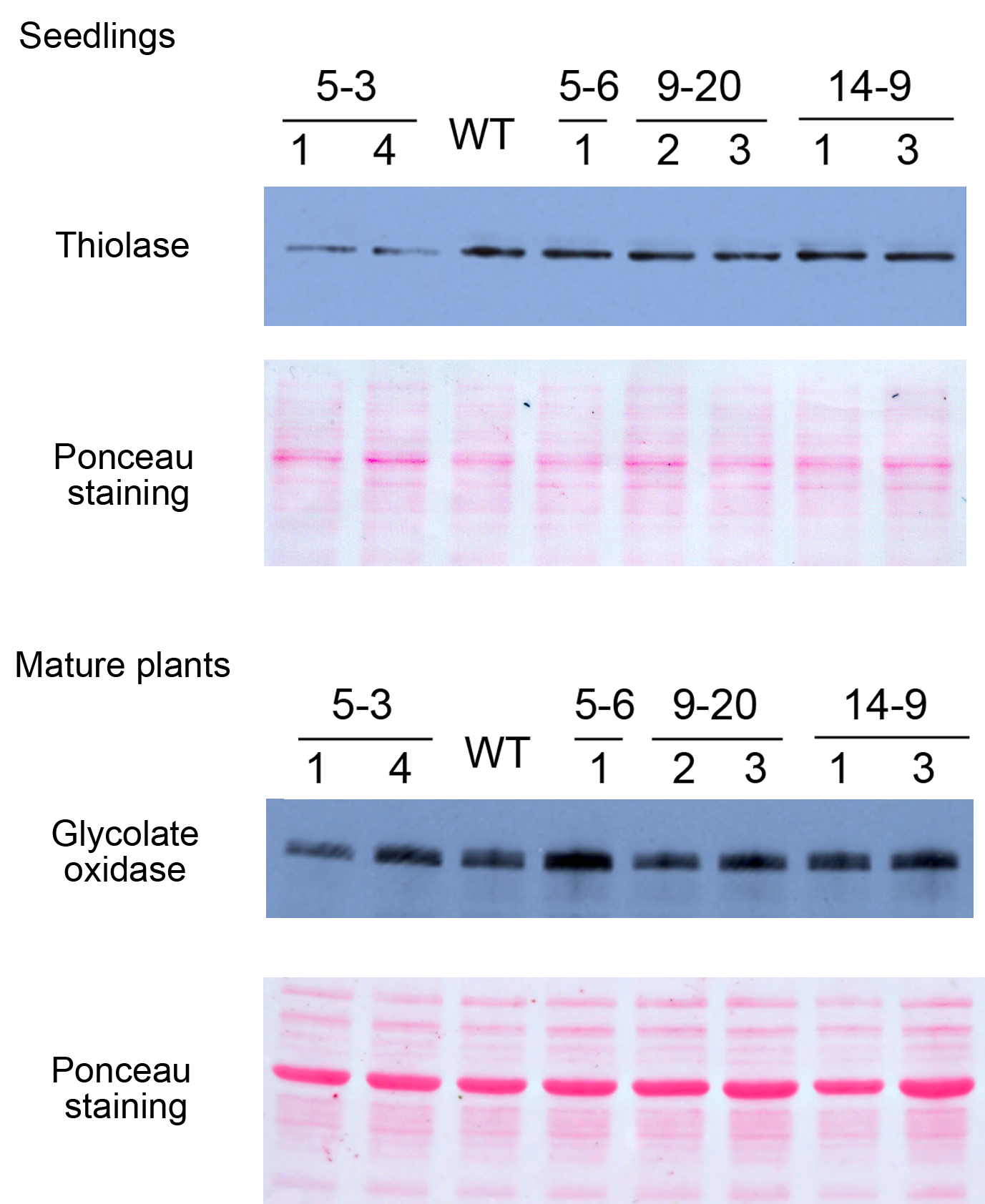

Supplement: Figure S3 — PEX11e transgenics do not have altered levels of glycolate oxidase or 3-ketoacyl thiolase protein. Western blot analysis of thiolase and glycolate oxidase in total protein extracts (20 µg per lane) from dark grown seedlings (thiolase) and green leaves of 4 week old plants (glycolate oxidase) of the indicated lines. The Ponceau S strained membrane is shown in each case to verify equal loading of the lanes. (6.98 MB TIF) [file pone.0009408.s003.tif]
